# Development and validation of a prognosis risk score model for preterm birth among pregnant women who had antenatal care visit, Northwest, Ethiopia, retrospective follow-up study

**DOI:** 10.1186/s12884-023-06018-1

**Published:** 2023-10-17

**Authors:** Bezawit Melak Fente, Mengstu Melkamu Asaye, Getayeneh Antehunegn Tesema, Temesgen Worku Gudayu

**Affiliations:** 1 Department of General Midwifery, School of Midwifery, College of Medicine & Health sciences, University of Gondar, Gondar, Ethiopia; 2 Department of Women’s and Family Health, School of midwifery, College of Medicine & Health sciences, University of Gondar, Gondar, Ethiopia; 3 Department of Epidemiology and Biostatistics, Institute of Public Health, College of Medicine and Health Sciences, University of Gondar, Gondar, Ethiopia; 4 Department of Clinical Midwifery, School of Midwifery, College of Medicine & Health sciences, University of Gondar, Gondar, Ethiopia

**Keywords:** Ethiopia, Pregnant women, Preterm birth, Prognosis model, Risk score

## Abstract

**Background:**

Prematurity is the leading cause of neonatal morbidity and mortality, specifically in low-resource settings. The majority of prematurity can be prevented if early interventions are implemented for high-risk pregnancies. Developing a prognosis risk score for preterm birth based on easily available predictors could support health professionals as a simple clinical tool in their decision-making. Therefore, the study aims to develop and validate a prognosis risk score model for preterm birth among pregnant women who had antenatal care visit at Debre Markos Comprehensive and Specialized Hospital, Ethiopia.

**Methods:**

A retrospective follow-up study was conducted among a total of 1,132 pregnant women. Client charts were selected using a simple random sampling technique. Data were extracted using structured checklist prepared in the Kobo Toolbox application and exported to STATA version 14 and R version 4.2.2 for data management and analysis. Stepwise backward multivariable analysis was done. A simplified risk prediction model was developed based on a binary logistic model, and the model’s performance was assessed by discrimination power and calibration. The internal validity of the model was evaluated by bootstrapping. Decision Curve Analysis was used to determine the clinical impact of the model.

**Result:**

The incidence of preterm birth was 10.9%. The developed risk score model comprised of six predictors that remained in the reduced multivariable logistic regression, including age < 20, late initiation of antenatal care, unplanned pregnancy, recent pregnancy complications, hemoglobin < 11 mg/dl, and multiparty, for a total score of 17. The discriminatory power of the model was 0.931, and the calibration test was p > 0.05. The optimal cut-off for classifying risks as low or high was 4. At this cut point, the sensitivity, specificity and accuracy is 91.0%, 82.1%, and 83.1%, respectively. It was internally validated and has an optimism of 0.003. The model was found to have clinical benefit.

**Conclusion:**

The developed risk-score has excellent discrimination performance and clinical benefit. It can be used in the clinical settings by healthcare providers for early detection, timely decision making, and improving care quality.

**Supplementary Information:**

The online version contains supplementary material available at 10.1186/s12884-023-06018-1.

## Introduction

Preterm babies are at a higher risk of morbidity and mortality than other segments of the population [1]. According to the World Health Organization (WHO), approximately 15 million babies are born prematurely worldwide each year, accounting for more than one-tenth of all babies born globally and nearly one million children die as a result of preterm birth complications [2]. Preterm birth rates range from 5 to 18% of all babies born in 184 countries [2]. Even though preterm birth is a worldwide issue, more than 60% of preterm births occur in South Asia and Africa, where preterm infants account for 46% of all neonatal deaths [3]. Every year in Ethiopia, 320,000 babies are born prematurely, with 24,400 children under the age of five dying as a result of direct preterm complications [1]. The neonatal mortality rate was 33 deaths per 1000 live births in 2019, according to the Ethiopian Mini-Demographic and Health Survey (EDHS), with prematurity and its associated complications being the leading cause [4].

Prematurity is the leading cause of newborn deaths worldwide [5] and, the second leading cause of all child deaths under the age of five, after pneumonia [6, 7]. The majority of preterm infants who survive have disabilities such as cerebral palsy, sensory deficits, learning disabilities, and respiratory problems. Preterm birth morbidity frequently persists into adulthood, putting the individual and family under physical, emotional, and financial strain [8–12].

Preterm birth risk factors are poorly understood. However, certain factors are known to increase a woman’s risk of having a preterm birth. Previous research found that maternal age, residence, marital status, Middle Upper Arm Circumference (MUAC), maternal weight, gravity, parity, number of antenatal care initiation(ANC) visits, hemoglobin(HBG) < 11 g/dl, pregnancy status, timing of ANC initiation, iron folate taken, inter-birth interval, medical illness, recent pregnancy complication, past bad obstetrics history, and previous mode of delivery were all significant predictors of preterm birth [12–31].

More than three-quarters of preterm babies can be saved with low-cost care [2], Quality care before, between, and during pregnancy were necessary to ensure all women had a positive pregnancy experience [32]. Such as counseling on a healthy diet and optimal nutrition, a minimum of eight contacts with health professionals throughout pregnancy to identify and manage other risk factors, essential care during childbirth, and antenatal steroid injections [2, 33]. Prognosis risk score models for preterm birth were developed in high-resource settings. However, the predictors they used, such as fetal fibronectin or cervical length screening, are not routinely done in low-resource settings. To meet the Sustainable Development Goal (SDG), all countries, including Ethiopia, plan to reduce neonatal mortality to 12 per 1,000 live births by 2030 [34]. In order to reduce neonatal and child mortality, addressing preterm birth a crucial issue. Thus, developing a prognosis risk score model for preterm birth based on demographic and clinical risk factors alone is very important in the context of Ethiopia. It will be incorporated into the routine ANC services for the detection and management of high-risk women for the prevention of preterm births. Also, for women at low risk, the score model will reduce unnecessary overtreatment and hospitalizations in the routine healthcare delivery system. Hence, the prognosis risk score may contribute in reducing avoidable neonatal morbidity and mortality which is related to prematurity at the local, regional, and national level. As a result, this study aimed to develop and validate a prognosis risk score model for preterm birth based on easily available factors.

## Methods and materials

### Aim

To develop and validate a prognosis risk score model for preterm birth among pregnant women who had antenatal care visit at Debre Markos Comprehensive and Specialized Hospital, Ethiopia.

### Study design

In this study, an institution-based retrospective follow-up study was conducted at Debre Markos Comprehensive and Specialized Hospital in Ethiopia to develop and validate a prognosis risk score model for preterm birth among pregnant women who had antenatal care visits.

The theoretical design [35] of the study was; the incidence of preterm birth (PTB) at a future time “t” is a function of multiple prognostic determinants measured at time point before the occurrence of preterm birth or during pregnancy, “t0”. The domain was pregnant women who had antenatal care.

PTB (t0 + 1) = f (X1(t0) + X2(t0) + X3(t0) + …).

The theoretical design of this study was found to be:

PTB (t_0 + 1_) = f (Age (t_0_) + Timing of ANC initiation (t_0_) + Pregnancy status (t_0_) + Recent Pregnancy complications (t_0_) + HGB (t_0_) + Parity (t_0_)).

Probability of preterm birth = f (predictor variables).

PR (preterm birth) = f (age, timing of ANC initiation, pregnancy status, recent pregnancy complications, HGB, parity).

PR (Y = 1) = f (X), where Y = 1 means having preterm birth and Y = 0 means not having preterm birth.

PR (Y = 1) = f (βo + β1 age + β2 timing ANC initiation + β3 pregnancy status + β4 + recent pregnancy complications + β5 HGB + β6 parity).

The study was conducted at Debre Markos Comprehensive and Specialized Hospital from January 1, 2020 to August 30, 2022, and data were extracted from January 3, 2023 to February 1, 2023.

The study population was all pregnant women who had ANC visits at Debre Markos Comprehensive and Specialized Hospital from January 1, 2020 to August 30, 2022. All pregnant women who had ANC follow-ups from January 1, 2020 to August 30, 2022, and gave birth at Debre Markos Comprehensive and Specialized Hospital were included. While, pregnant women who had neither certain Last Menstrual Period (LMP) nor early ultrasound (U/S) evidences and twin pregnancy were excluded.

### Sample size and sampling technique

Sample size calculation using rule of thumb. As a general rule, the required sample size in the development of a prediction model is at least 10 events per predictor [36]. We considered 12 predictors with easily accessible and significant clinical or statistical effects for preterm birth obtained from pooled associated factors of preterm birth systematic reviews and meta-analysis studies in Ethiopia [28, 37]. With incidence of preterm birth is 11.4% [37].

N= (n*10)/I, (12*10)/0.114 = 1,053 with 10% missing = **1,160**.

The samples were then chosen using simple random sampling with ANC and delivery registration logbooks. During the study period, 3,423 women received antenatal care at DMCSH. The total number of pregnant women who received antenatal care and gave birth at the hospital was 2,690. The computer-generated simple random sampling procedure was used to select **1,160** records (charts) of study participants.

### Variables of the study

#### Dependent variable

Preterm birth (Yes/ No).

### Independent variables

#### Socio-demographic factors

Age, marital status, MUAC, maternal weight, and place of residence.

#### Antenatal characteristics

Gravity, parity, hemoglobin, pregnancy status, timing of ANC initiation, number of ANC visit, the timing of the start of iron folate, and inter-birth interval.

#### Medical illness like

UTI, HIV/AIDS, chronic hypertension, diabetes mellitus, and malaria.

#### Recent pregnancy complication

Antepartum hemorrhage, PROM, PIH, gestational diabetic mellitus, and threatened abortion.

### Operational definitions

#### Preterm birth

Defined as the birth of a neonate at a gestational age greater than 28 weeks but less than 37 weeks [38]. Duration of pregnancy was dichotomized as “Yes” for preterm birth if it occurred between 28 weeks and 36 + 6 weeks of gestational age, and “No” if it occurred after 37 weeks of gestational age.

GA was calculated using a certain Last Menstrual Period (LMP) date and/or an established early pregnancy ultrasound (U/S) date (up to and including 20 completed weeks of gestation). When the LMP and U/S dates were not correlated, the American College of Obstetricians and Gynecologists (ACOG) recommendation required defaulting to U/S for GA assessment [39].

#### Discrimination

Performance of model to differentiates pregnant women who give and who did not give preterm birth. AUROC is > 0.9 excellent discrimination ability [40].

#### Calibration

the agreement between observed proportions of preterm birth and predicted probabilities preterm birth. Well calibration mean calibration plot show over a 45° line and Hosmer–Lemeshow test statistics which is insignificant (p > 0.05) [41].

#### Late initiation of antenatal care

Pregnant women who start antenatal care visit after 12 weeks of gestation [42].

#### Risk score

are tools that combine multiple predictors by assigning relative weights to each predictor to obtain a risk or probability of a condition happening. It is simple to calculate, easily interpretable, and actionable [43].

### Data collection procedure and tool

A data extraction checklist was prepared on the Kobo Toolbox web-based tool for the collection of data from the mother’s medical records. A modification was made after reviewing some patients’ records. The checklist was arranged into socio-demographic characteristics of the pregnant mothers, medical illnesses, past and recent obstetric characteristics, and birth outcomes. The data were collected by four BSc midwives from January 3, 2023 to February 1, 2023.

### Data quality control and assurance

After reviewing the prior literature, taking suggestions from medical experts, and reviewing more than 40 charts of the study participant’s, a data extraction checklist was developed in English for data collection. After reviewing the charts, I added MUAC and maternal weight. Data collectors were trained for two days by the principal investigator before the actual data collection about the Kobo Toolbox and components of the checklist, the sequence, and how to solve potential problems they may face during data collection time. Frequent and timely supervision of data collectors was undertaken. The collected data were checked for completeness and accuracy during data collection by the principal investigator.

### Data processing and analysis

Data were collected using Kobo Toolbox. The collected data were exported to STATA version 14 and R Software version 4.2.2 for data management and analysis. Missing data were handled by multiple imputation, which predict the missing values by utilizing the existing information and then substituting the missing values with the predicted values to create a complete dataset with regression by assuming missing values at random. A sensitivity analysis was done (Table 1). Predictors imputed include hemoglobin level, maternal weight, MUAC, and pregnancy status, with missing values of 46 (4.06%), 22 (1.94%), 48 (4.24%), and 52 (4.41%), respectively. Multi-collinearity among independent predictors was checked by the variance inflation factor (VIF).

**Table 1:** Sensitivity analysis of the model to predict preterm birth: Comparison of the regression coefficients, standard errors (SE), and p-values for complete case analysis (CCA) and multiple imputed data (MI).

Predictor Variable	Complete Case Analysis			Multiple Imputation		
	β	SE	P-value	β	SE.	P-value
Age (< 20)	1.656	0.669	0.013	1.62	0.648	0.012
Parity (multipara)	1.067	0.431	0.013	1.31	0.424	< 0.001*
Recent pregnancy complications(Yes)	3.469	0.31	< 0.001	3.61	0.304	< 0.001*
Time of ANC initiation( Late)	0.763	0.385	0.047	0.78	0.383	0.032*
Comorbid(Yes)	0.265	0.924	0.774	0.28	0.924	0.762
Pregnancy status(unplanned)	1.309	0.297	< 0.001	1.18	0.288	< 0.001*
Weight (underweight)	0.312	0.439	0.477	0.32	0.441	0.461
MUAC(< 24 cm)	0.623	0.375	0.097	0.67	0.371	0.069*
Hemoglobin(< 11 g/dl)	4.171	0.482	< 0.001	4.19	0.482	< 0.001*

*Variables retained in the reduced model using the likelihood ratio test are: age, timing ANC initiation, pregnancy status, hemoglobin, parity, and recent pregnancy complications. Recent pregnancy complications include antepartum hemorrhage, PROM (premature rupture of membrane), PIH (pregnancy-induced hypertension), gestational diabetic mellitus, and threatened abortion. *ANC* Antenatal care

Transparent Reporting of a multivariable Prediction model for Individual Prognosis Or Diagnosis (TRIPOD) guideline is used for developing and reporting prediction model [44]. Tables and figures were used to describe the characteristics of the study participants. Categorical variables were described by frequencies and percentages. Continuous variables were presented as mean/medians and standard deviation/interquartile ranges (IQR) depending upon the distribution of the variable.

Logistic regression analysis was used to evaluate which variables are most powerful in predicting preterm birth [40]. A bivariable regression analysis was used to obtain insight into the association of each potential determinant with preterm birth and for inclusion in a multivariable regression analysis. Variables with a p-value ≤ 0.25 in the bivariable analysis were fitted to the multivariable regression analysis. After a stepwise backward elimination technique was used, the role of each predictor in the multivariable analysis was assessed by the likelihood ratio test. To be more liberal, a p-value ≤ 0.15 for the likelihood ratio test was used to fit the reduced model [43]. The preterm birth risk score model was developed from significant variables in the reduced multivariable regression model. The regression coefficients of the reduced model were used as a measure of the effect of predictor variables on the probability of preterm birth.

The theoretical design of the study was: the incidence of preterm birth at a future time “t” is a function of prognostic determinants such as socio-demographic, previous obstetric, and recent pregnancy-related factors measured at one or more time points before the occurrence of preterm births, “t_0_”, which is the moment of prognostication. It is written in the following way:

PTB (t_0 + 1_) = f (Age (t_0_) + Timing of ANC initiation (t_0_) + Pregnancy status (t_0_) + Recent Pregnancy complications (t_0_) + HGB (t_0_) + Parity (t_0_)).

The risk score was developed using identified coefficients, for which the weights were defined as the quotient of the corresponding estimated coefficient from a reduced multivariable regression analysis divided by the smallest beta coefficient. The number of points was subsequently rounded to the nearest integer. We determined the total score for each individual by assigning points for each variable present and adding them up. The score was dichotomized, allowing each pregnant woman to be classified as having a high or low-risk of preterm birth. The model’s performance was assessed using discrimination power, calibration, Brier score, and prediction density plot. The area under the receiver operating characteristic curve was used to evaluate the discrimination power of the individual predictors and developed a prediction model. AUROC is < 0.5 have no information, between 0.5 and 0.7 poor, between 0.7 and 0.9 good, and > 0.9 excellent discrimination ability, and 1 is the ideal one which is perfect discrimination [43]. A ROC curve is used to assess the performance of a categorical classifier by “ROCit” and ‘‘pROC’’ packages of R software with plot of sensitivity (true positive rate) versus 1-specificity (false positive rate). Model calibration was assessed using a calibration plot and p-value to ensure the reliability of the prediction models using the “givitiR” package in “R” software. The Brier score is also commonly used to assess performance for models that predict a binary outcome. The Brier score compares the squared differences between actual and predicted binary outcomes, with scores ranging from 0 for a perfect model to 0.25 [43].

The Youden index method [45] was used to determine the optimal cut-off point for categorizing pregnant women’s risk scores as high or low. Sensitivities, specificities, PPV, NPV, positive likelihood ratio, negative likelihood ratio, and accuracy were used to assess the predictive efficiency of the optimal cut-off values of the model. Internal validation was performed using the bootstrap procedure, which replicated the sample 2,000 times estimate how successfully the prediction model developed on the development set would perform on a hypothetical set of new patients. The clinical benefit of the prediction model was evaluated using decision curve analysis.

## Results

### Socio-demographic characteristics of the study participants

A total of 1,132 pregnant women were included in the study. The median age of mothers was 28, with an Interquartile Range (IQR) of 25 to 30 years, and 43.5% were between the ages of 25 and 29. More than three-quarters of the mothers lived in urban, and nearly all (97.35%) were married (Table 2).

**Table 2:** Socio-demographic characteristics of pregnant women who had ANC visits at DMCSH, Ethiopia, 2020–2022 (n = 1,132)

Variable	Category	Frequency	Percent
Age of mother at time of pregnancy	<20	39	3.45
	20–24	235	20.76
	25–29	493	43.55
	30–34	231	20.41
	≥35	134	11.84
Residence	Urban	986	87.10
	Rural	146	12.90
Marital status	Single	21	1.86
	Married	1102	97.35
	Divorced/widowed/Separated	9	0.80
MUAC	≥24	870	76.86
	<24 cm	262	23.14
Maternal weight	< 50 kg	196	17.31
	≥ 50 kg	936	82.69

*MUAC* Middle Upper Arm Circumference

### Maternal obstetrics history

Almost two-thirds of the pregnant women were multigravida. About 150 (13.25%) of the mothers had recent pregnancy complications, which 54 (36.24%) had APH followed by PROM 52 (34.67%) and PIH 50 (33.33%) (Table 3).

**Table 3:** Maternal obstetrics history of pregnant women who had ANC visit at DMCSH, Ethiopia, 2020–2022 (n = 1,132)

Variable	Category	Frequency	Percent
Gravidity	Primigravida	410	36.22
	Multigravida	722	63.78
Parity	Nullpara	462	40.81
	Primipara	313	27.65
	Multipara	357	31.54
Recent pregnancy complications	No	982	86.75
	Yes	150	13.25
Types recent pregnancy complications	Antepartum hemorrhage	54	36.24
	PROM	52	34.67
	Preeclampsia	50	33.33
	Threatened miscarriage	6	4.00
	Gestational hypertension	5	3.33
	Eclampsia	2	1.34

*SVD* spontaneous vaginal delivery, *PROM* Premature Rupture of Membrane

### Medical condition of mother during pregnancy

In this study, about 2.12% of the participants had medical illness during their recent pregnancy. Of them, about 9 (37.7%), 8 (33.3%) and 8 (33.3%) of the mothers had chronic hypertension, malaria and urinary tract infections, respectively. About 55 (4.86%) of the mothers were sero-positive for HIV (Table 4).

**Table 4:** Medical illness of pregnant women who had ANC visits at DMGCSH, 2020–2022

Variable	Category	Frequency	Percent
Medical illness	No	1108	97.88
	Yes	24	2.12
Types of medical illness	Chronic hypertension	9	37.5
	Malaria during current pregnancy	8	33.3
	UTI	8	33.3
	Diabetes mellitus	6	25
	Cardiac illness	1	4.7
	Syphilis	3	13%
HIV tested result	Negative	1077	95.14
	Positive	55	4.86

*HIV* Human Immune Deficiency Virus, *UTI* Urinary Tract Infection

### Antenatal characteristics of pregnant women

Nearly two-thirds (66.4%) of the pregnancies were planned, and only a quarter (25%) of the mothers had an early initiation of an ANC visit during their pregnancy. More than half (54.06%) of the mothers had four or more ANC visits for a recent pregnancy. The majority (73.59%) of the mothers initiated iron and folate supplementation in the second trimester of their pregnancy. About 52 (4.6%) of the mothers had low hemoglobin levels, and about 7.16% of participants were negative for the Rh factor (Table 5).

**Table 5:** Antenatal characteristics of pregnant women in their recent pregnancy who attend ANC visits at the DMGCSH, 2020–2022 (n = 1,132)

Variable	Category	Frequency	Percent
Pregnancy status	Planned	752	66.43
	Unplanned	380	33.57
Timing ANC initiation	Early initiation	285	25.18
	Late initiation	847	74.82
Number of ANC visits	< 4 visits	520	45.94
	≥ 4 visits	612	54.06
Time initiating iron	1st trimester	266	23.50
	2nd trimester	833	73.59
	3rd trimester	33	2.92
Hemoglobin	≥ 11 g/dl	1080	95.41
	< 11 g/dl	52	4.59
Rh factor	Positive	1051	92.84
	Negative	81	7.16

*Rh factor* Rhesus factor, *ANC* Antenatal care

### Prognosis model development and validation for preterm birth

#### Predictor selection for prediction of preterm birth

The incidence of preterm birth was 10.9% (95% CI: 9.2%, 12.8%). Demographic, medical, recent antenatal, and pregnancy-related conditions of mothers were considered for the development of a prognosis risk score model for preterm birth. In the bivariable binary logistic regression analysis (Supplementary Table 1) maternal age, residence, MUAC, maternal weight, gravidity, parity, the timing of ANC invitation, pregnancy status, hemoglobin level, medical illness, and recent pregnancy complications had a p-value ≤ 0.25 and were considered for the multivariable binary logistic regression analysis. However, underlying medical illness, residence, gravidity, and maternal weight were excluded in multivariable binary logistic regression analysis using the likelihood ratio test at a p-value > 0.15 (Table 6).

**Table 6:** Multivariable binary logistic regression coefficients and risk score for variables retained in the final reduced model for prediction of preterm birth among pregnant women attending ANC, 2020–2022

Predictor Variable	category	Multivariable Analysis		Reduced model		Risk Score
		β (95%CI)	p-value	β (95%CI)	p-value	
Age	< 20	1.458 (0.124–2.791)	0.032	1.628(0.363, 2.892)	0.012	2
	20–24	1		1		
	25–29	0.077 (-0.86-1.014)	0.872	0.114(-0.792, 1.02)	0.805	
	30–34	-0.009(-0.754-0.736)	0.98	-0.011(-0.74,0.724)	0.977	
	≥ 35	− 0.715 (-1.639-0.209)	0.129	-0.642(-1.55,0.266)	0.166	
Residence	Urban	1				
	Rural	0.349 (-0.347-1.044)	0.326	NA		
MUAC	≥ 24 cm	1		1		
	< 24 cm	0.616 (-0.122-1.353)	0.102	0.523(-0.096,1.142)	0.098	
Weight of Mother	≥ 50 kg	1				
	< 50 kg	-0.33(-1.201.542)	0.458	NA		
Gravidity	Primigravida	1				
	Multigravida	-0.337(-1.689-1.015)	0.625	NA		
Parity	Nullpara	1		1		
	Primipara	0.782 (-0.497-2.061)	0.231	0.565(-0.252,1.382)	0.175	
	Multipara	1.359 (0.086–2.632)	0.036	1.138(0.324,1.953)	0.006	2
Timing of ANC	Early initiation	1		1		
	Late initiation	0.821 (0.062–1.58)	0.034	0.782(0.042,1.522)	0.038	1
Pregnancy status	Unplanned	1.194 (0.622–1.766)	0.000	1.188(0.626,1.751)	0.001	2
	Planned	1		1		
Hemoglobin	≥ 11 g/dl	1		1		
	< 11 g/dl	4.227 (3.268–5.185)	0.000	4.198(3.254,5.141)	0.001	5
Comorbidity	No	1				
	Yes	0.273 (-1.548-2.094)	0.769	NA		
Recent pregnancy complication	No	1		1		
	Yes	3.59 (2.994–4.186)	0.000	3.613(3.019,4.207)	0.001	5
Constant		-5.627(-6.702-4.552)		-5.682(-6.71-,4.64)	17 total risk score	

*MUAC* Middle Upper Arc Circumference, *ANC* Antenatal care, *CI* Confidence Interval

### Model development and prediction model performance assessment

**Risk prediction model = -**5.68 + 1.62 age + 0.78 timing of ANC initiation + 1.18 pregnancy status + 3.61 recent pregnancy complications + 4.19 HGB + 1.31 parity.

The equation provided above estimates the probability of preterm birth based on the status of the predictors.

Individual predictors in the final reduced model have low performance, then the final reduced model discriminates the risk of preterm birth among pregnant women with an AUROC ranging from 0.53 to 0.80. But they had good discriminating abilities in combination (Table 7).

**Table 7:** AUROC of individual and combined predictors in the final reduced model for predicting preterm birth among pregnant women who attend ANC visits at DMCSH, 2020–2022

Predictors	AUROC (95%CI)
Age	0.53( 0.47–0.59)
Timing ANC initiation	0.58(0.55–0.61)
Pregnancy status	0.70( 0.66–0.74)
Parity	0.58(0.53–0.64)
HGB	0.65(0.61–0.69)
Recent pregnancy complication	0.80(0.76–0.84)
Age + Recent pregnancy complication	0.83( 0.78–0.87)
Age + recent pregnancy complication + HGB	0.89(0.86–0.93)
Age + Timing ANC initiation + Pregnancy status + HGB	0.82(0.77–0.86)
Age + Timing ANC initiation + Pregnancy status + HGB + Parity	0.83(0.78–0.87)
Age + recent pregnancy complication + Timing ANC initiation + Pregnancy status + Parity	0.88( 0.84–0.92)

**Fig. 1:**
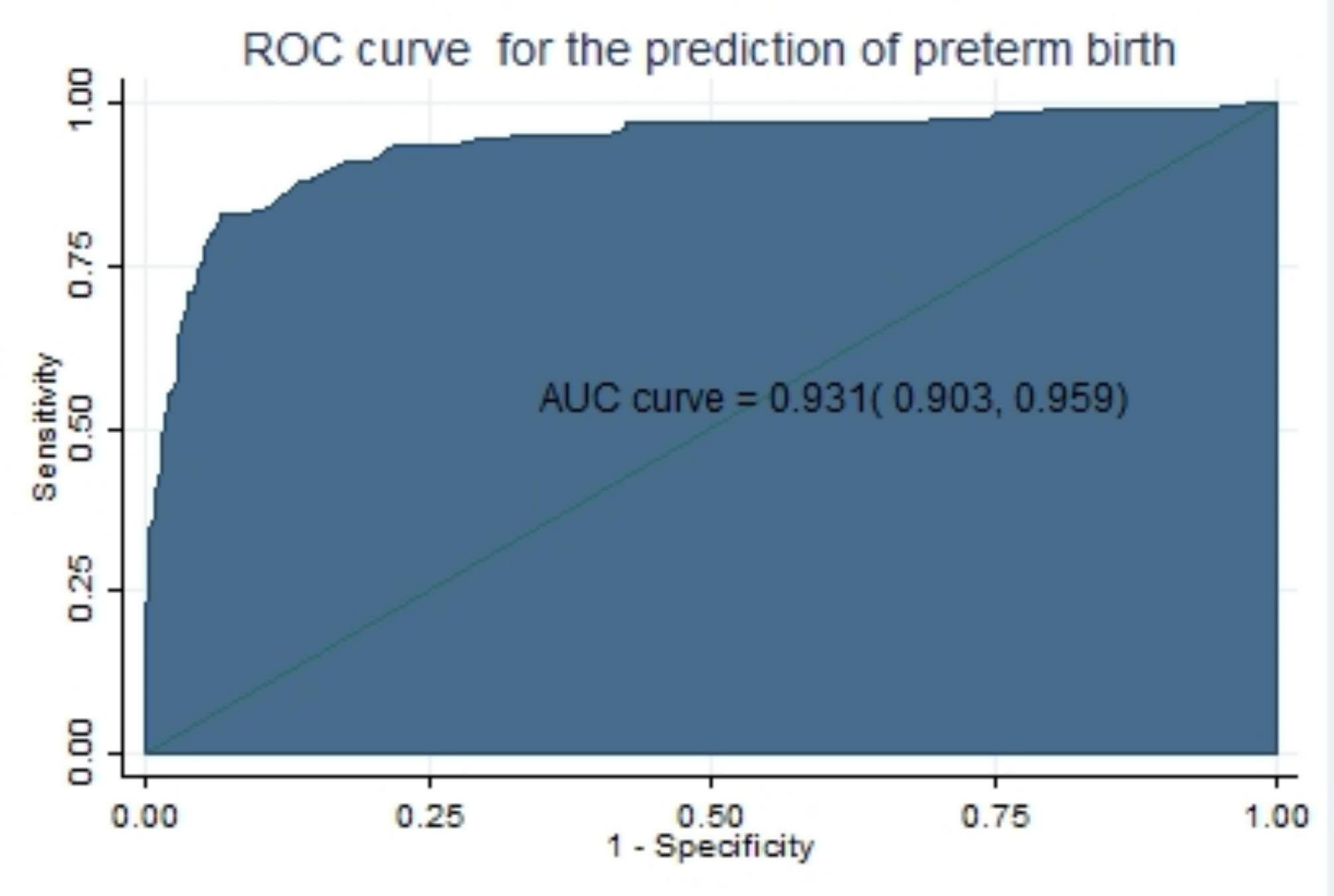
The ROC curve represents the probability of risk for preterm birth, DMCSH, 2020–2022

Finally, the area under the ROC of the final reduced model using six predictors was 0.931 (95% CI: 0.903, 0.959) using original beta coefficients, which means a model was 93.1% differentiates pregnant women who give and do not give preterm birth (Fig. 1).

**Fig. 2:**
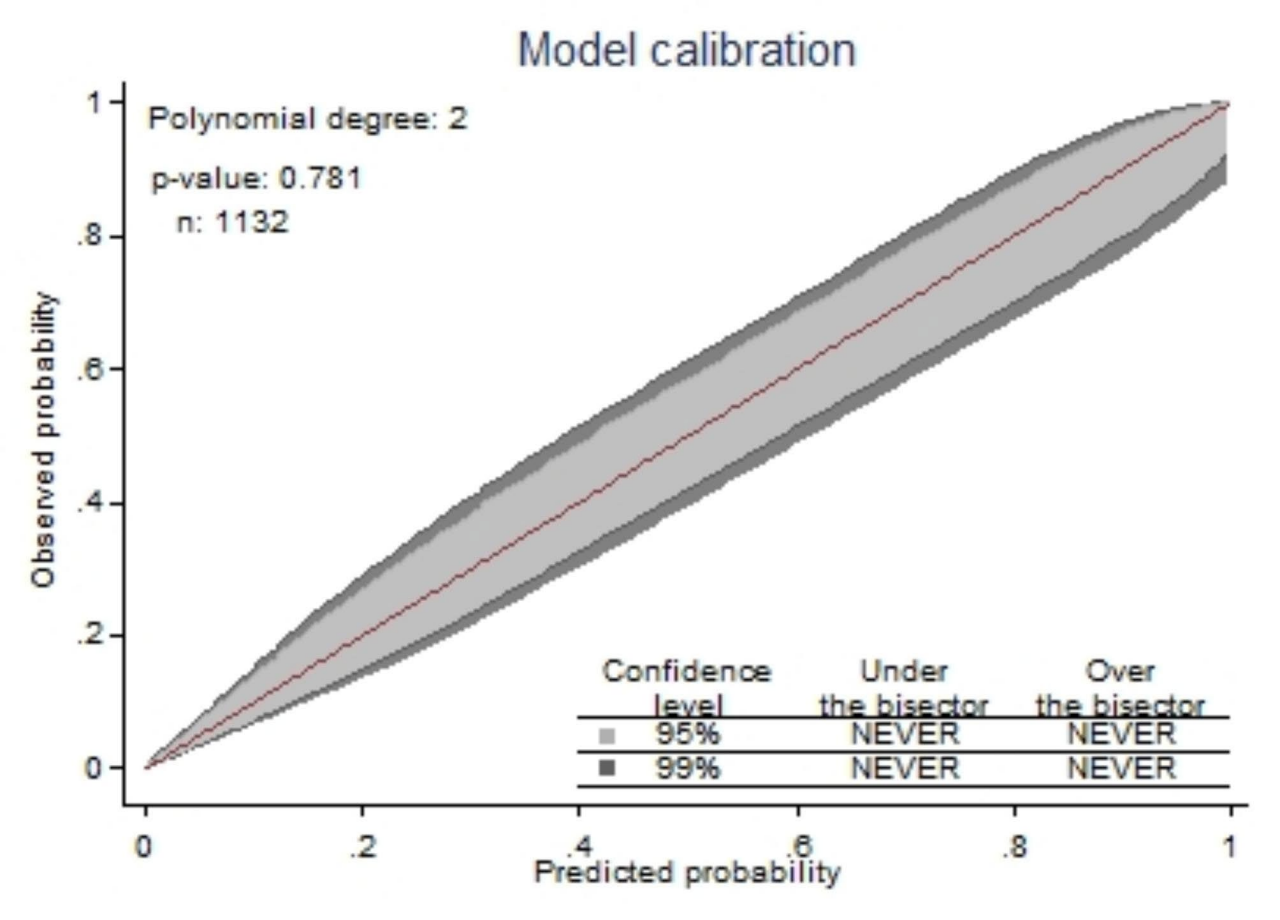
Calibration plot for developed model, DMCSH, 2020–2022

The developed model was well calibrated (p = 0.781) and calibration plot falling along a 45° line (Fig. 2) or insignificant statistical test by Hosmer-Lemshow (p-value = 0.066), this indicates that the model well represented the data (there was agreement between observed outcomes and the predicted probability).

The developed model had excellent performance based on a Brier score of 0.0478 and an approach of 0. The perfect prediction model has a 0 Brier score, and near one has poor accuracy, with scores ranging from 0 to 0.25 is a perfect model.

### Prediction density plot

**Fig. 3:**
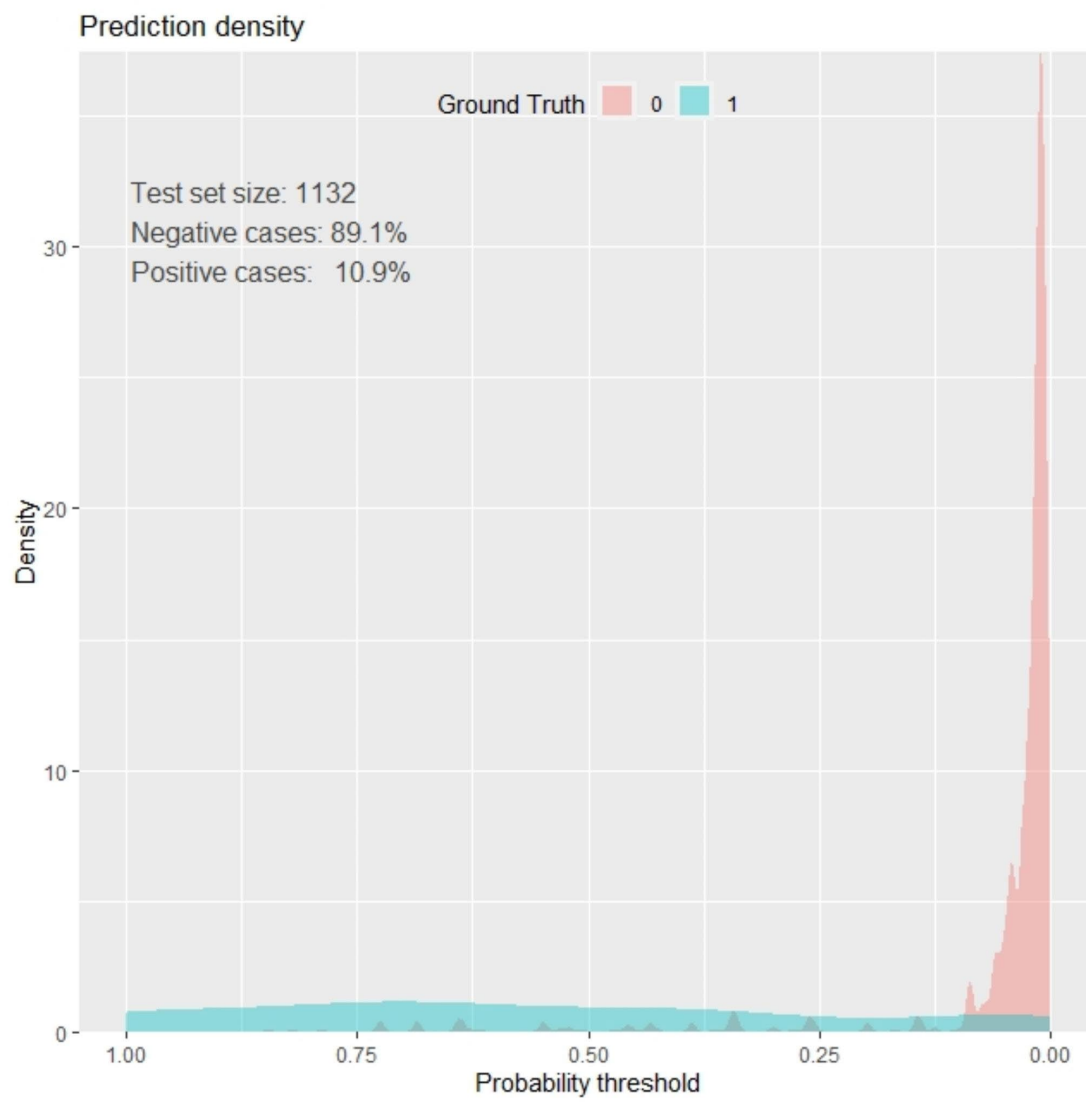
Prediction density plot for developed model using original beta coefficients at DMCSH, 2020–2022

The model’s ability to classify pregnant women with preterm and term births was also assessed in terms of the prediction density plot. There was overlapping between non cases labeled red (mothers without preterm birth) and positive cases labeled green (mothers with preterm birth) along the threshold probability. So, our model is not 100% accurate in predicting or identifying the difference between pregnant women who have preterm births and those who do not (Fig. 3).

### Cutoff point for probability of preterm birth

The optimal cutoff point for the predicted probability of the risk of preterm birth was 0.2125, with the sensitivity, specificity, PPV, and NPV were 82.9%, 93.3%, 60.0%, and 97.8%, respectively, using the beta coefficients of the predictor variables. The accuracy was also 92.1% (Table 8).

**Table 8:** Performance of the prediction model for preterm birth based on original beta coefficients at different cut-off points for pregnant women who had ANC at DMCSH, 2020–2022

Cut point	Sensitivity	Specificity	PPV	NPV	Accuracy	LR+	LR-
(≥ 0 0.1255)	82.93%	91.28%	53.68%	97.77%	90.37%	9.5083	0.1870
**(≥ 0 0.2125 )**	**82.93%**	**93.26%**	**60.00%**	**97.82%**	**92.14%**	**12.3049**	**0.1831**
(≥ 0 0.3243 )	76.42%	94.75%	63.95%	97.06%	92.76%	14.5492	0.2488
(≥ 0 0.4545 )	68.29%	96.33%	69.42%	96.14%	93.29%	18.6236	0.3291
(≥ 0.5234)	55.28%	97.62%	73.91%	94.71%	93.02%	23.2425	0.4580

*LR+* Positive Likelihood Ratio, *LR* Negative Likelihood Ratio, *NPV* Negative Predictive Value, *PPV* Positive Predictive Value

### Risk score development using simplified risk score

For simplicity of clinical use and to avoid sophisticated risk calculation, a simplified risk score prediction model for the estimated risk of preterm birth was developed by rounding the regression coefficients to the nearest integer after weighting with the least coefficient.

The estimated risk score of preterm birth was calculated as:

Risk prediction model = -5.68 + 1.62 age + 0.78 timing of ANC initiation + 1.18 pregnancy status + 3.61 recent pregnancy complications + 4.19 HGB + 1.31 parity

Simplified risk score (preterm birth) = 2 *Age (< 20) + 1 *Timing ANC initiation (late) + 2 *Pregnancy status (unplanned) + 5 *Recent pregnancy complication (yes) + 5 *HGB (< 11) + 2 *Parity (multipara).

**Fig. 4:**
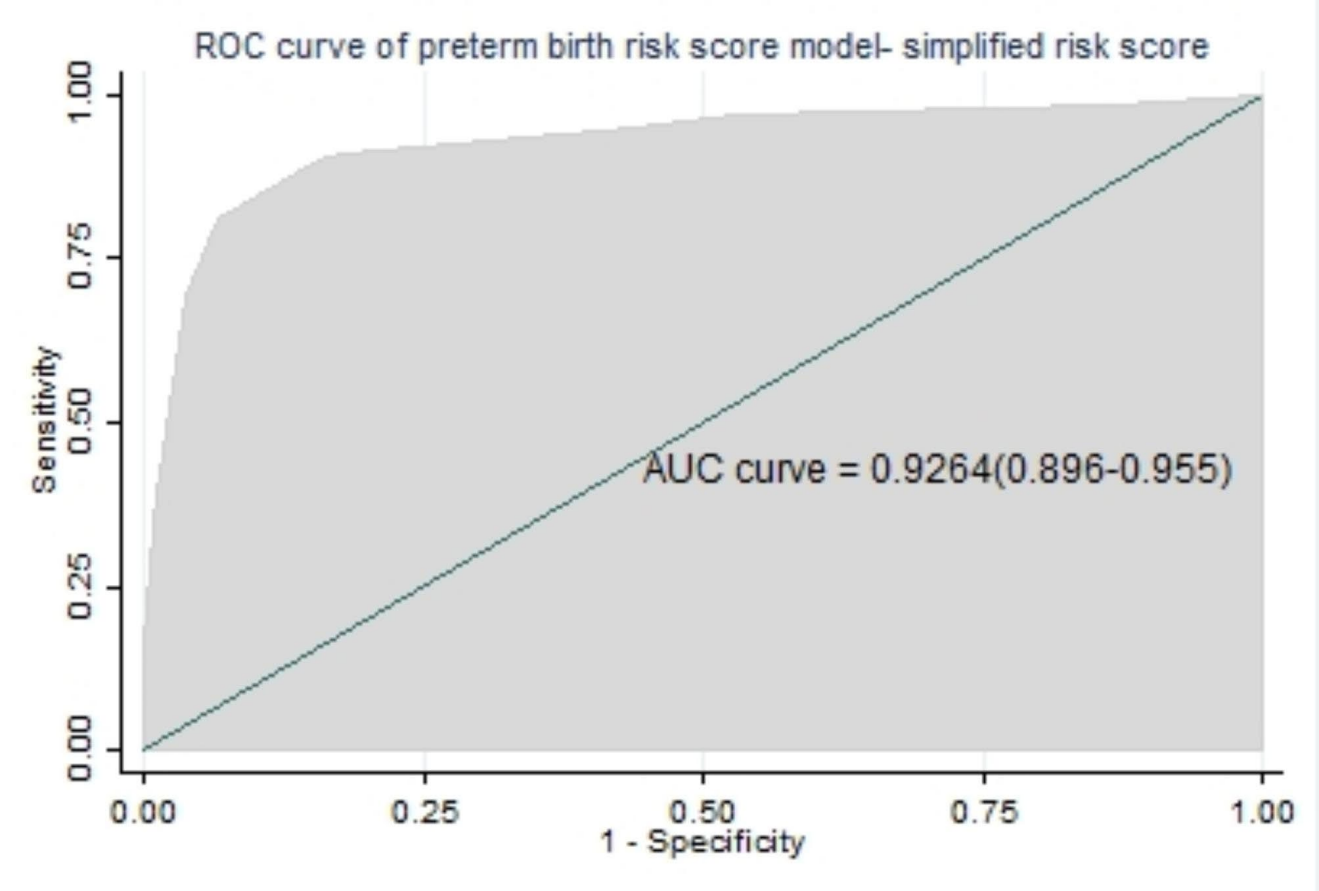
Area under the ROC curve for preterm birth using simplified risk score

All significant regression coefficients in the final reduced model were used for model development using a simplified risk score. The AUROC in the simplified risk score was 92.6% (89.7-95.6%) (Fig. 4), and the calibration had a p-value of 0.793. For the sake of simplicity, little improvement, and ease to use, we preferred a simplified risk score from the original beta coefficient for prediction model development.

#### Risk classification for preterm birth using simplified risk score

The optimal cutoff point was suggested by the Youden index [45] and finally, pregnant women who score lower than 4 were classified as low risk, and pregnant women who score 4 and above were classified as higher risk for preterm birth. When we dichotomize high-risk (≥ 4) and low-risk (< 4) based on risk scores, 292 (25.8%) of the pregnant mothers were at high risk and 840 (74.2%) were at low risk for preterm birth (Table 9). The sensitivity of 91.1%, specificity of 82.2%, NPV of 98.6%, PPV of 85.2%, and accuracy of 83.1% of the risk scores at the optimal cutoff 4 and with a likelihood ratio positive of 5.104 and a likelihood ratio negative of 0.108 (Table 10).

**Table 9:** Risk classification for preterm birth based on simplified risk score (n = 1,132) among pregnant women who had ANC at DMCSH, 2020–2022

Risk group	Classification		Incidence of preterm birth	
	No. of mothers	%	No. of mothers	%
**Low(< 4)**	840	74.2	11	1.1
**High(≥ 4)**	292	25.8	112	9.8
**Total**	1,132	100	123	10.9

**Table 10:** Performance of risk score at different cutoff point for preterm birth among pregnant women attending antenatal care at DMCSH, 2020 to 2022

Cut point	Sensitivity (%)	Specificity (%)	Accuracy (%)	NPV (%)	PPV (%)	LR+	LR-
(≥ 1 )	98.3	13.2	22.5	98.5	12.1	1.1344	0.1224
(≥ 2 )	96.7	47.1	52.5	99.1	22.2	1.8315	0.0689
(≥ 3 )	95.1	56.1	60.4	98.9	25.9	2.1714	0.0868
**(≥ 4 )**	**91.0**	**82.2**	**83.1**	**98.6**	**85.2**	**5.1042**	**0.1088**
(≥ 5 )	90.2	84.3	84.9	97.6	87.4	5.7630	0.1157
(≥ 6 )	81.3	93.4	92.1	96.5	89.1	12.4292	0.2001
(≥ 7 )	71.5	95.5	92.9	96.5	90.0	16.0419	0.2978
(≥ 8 )	69.1	96.2	93.2	90.6	91.7	18.3494	0.3210
(≥ 9 )	42.2	98.4	92.3	89.3	92.1	26.6605	0.5865

*LR+* Positive Likelihood Ratio, *LR* Negative Likelihood Ratio, *NPV* Negative Predictive Value, *PPV* Positive Predictive Value

Developed prediction models using original beta and simplified risk score had similar discrimination ability as well as comparable sensitivity and specificity for their optimal cut-off points. The possible minimum and maximum score was 0 and 17. According to the developed risk score, of all the mothers included in the study 840 (74.2%) were categorized under low-risk group and the proportion of preterm birth were 1.1%. Two hundred ninety two mothers found to be high risk with the percentage of preterm birth of 9.8% (Table 9).

### Internal validation

To check the optimism and bias of the developed model, the bootstrap technique was used for validating the model 2,000 random bootstrap samples with replacement were drawn from the data set, with complete data on all predictors. The model’s predictive performance after bootstrapping is considered the performance that can be expected when the model is applied to similar future populations. The optimism correction estimate was also calculated by actual performance minus predicted performance, which was 0.003.

**Fig. 5:**
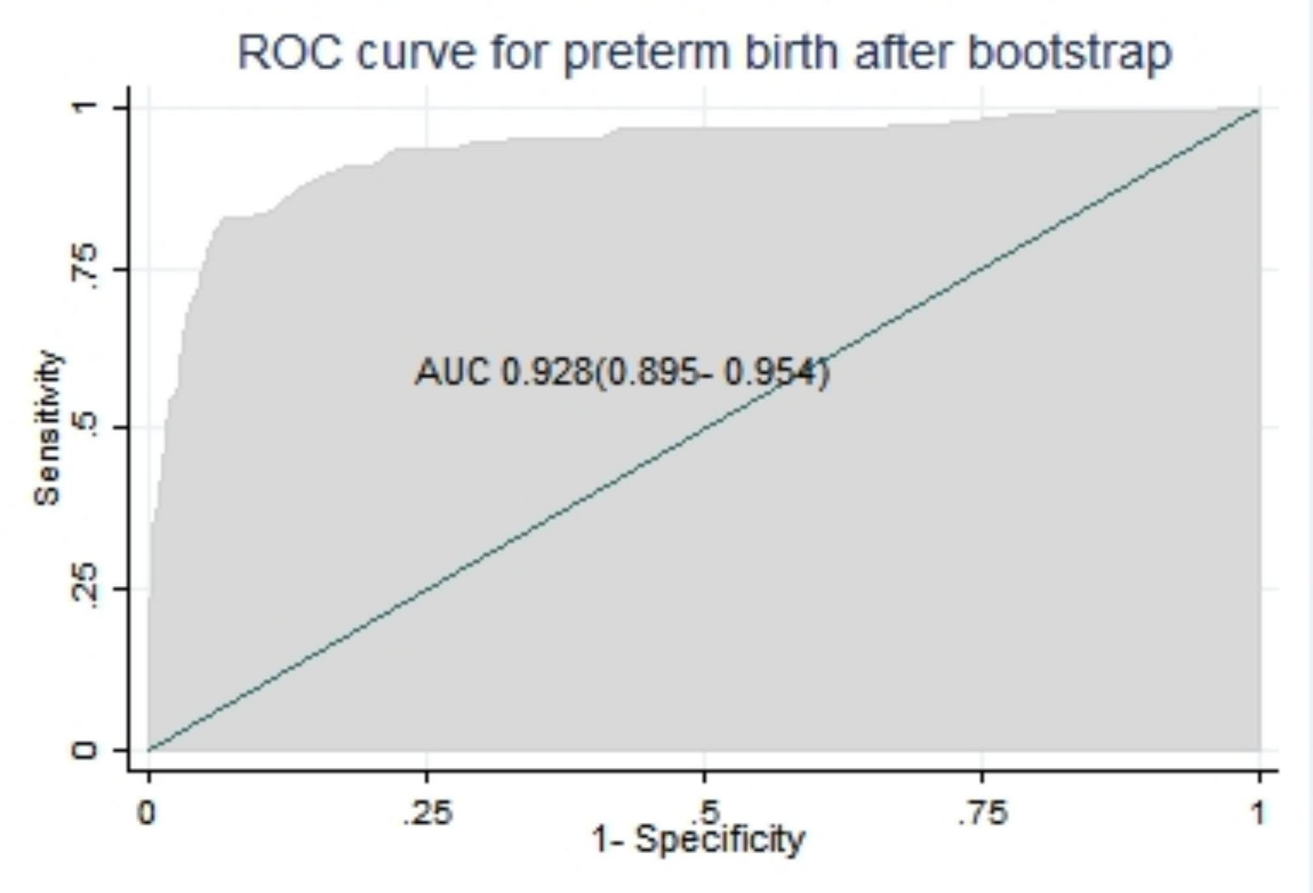
Area under the ROC curve for the risk score model for bootstrapped sample, DMCSH, 2020–2022

The bootstrap statistics show the bias in each original beta coefficient of the predictor in the prognosis model (Table 9). After subtracting the bias from each estimate, the discrimination power was again assessed. The bias-corrected beta-coefficients showed an AUC of 0.928 (95% CI: 0.895–0.954), which is similar performance to the model before the internal validation with an optimism coefficient (pooled bias) of 0.000243 (0.892–0.957) (Fig. 5).

#### Decision curve analysis

Provided the aim of developing this risk score model is for early differentiation of those who will be preterm birth so that they will be given critical attention to receive better treatment and appropriate care available. Besides model performance was assessed by AUC, sensitivity, specificity, and accuracy, clinical and public health utility of the model was also assessed by decision curve analysis (DCA) [40].

**Fig. 6:**
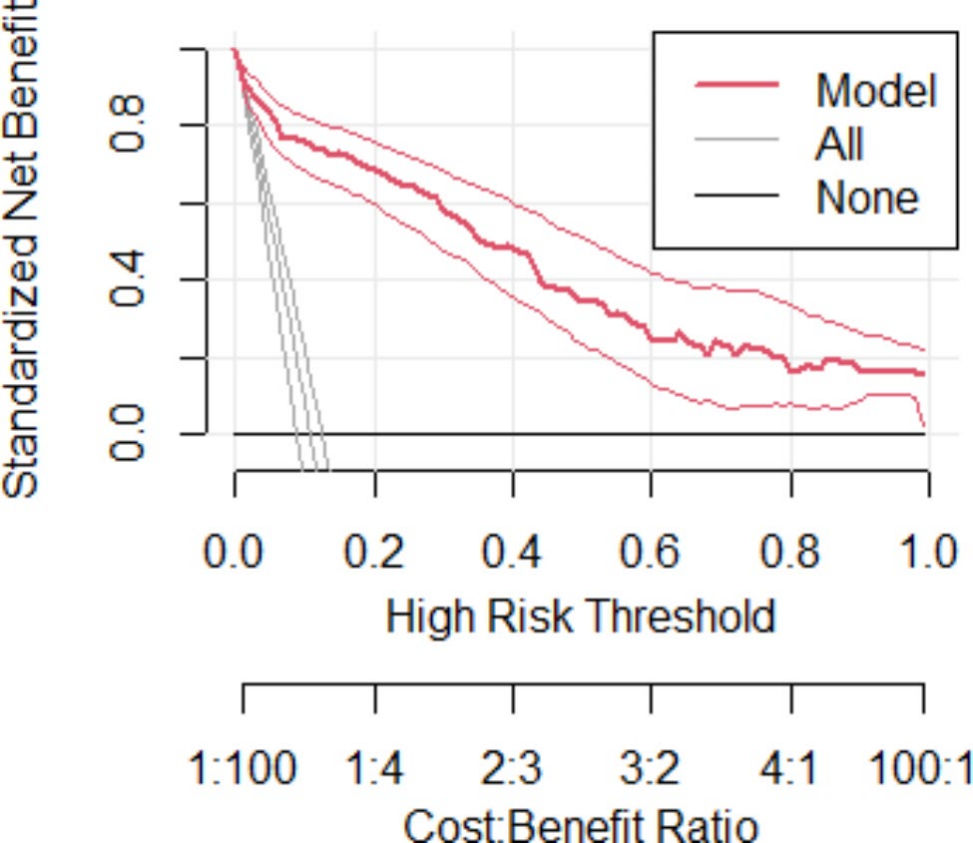
Decision curve analysis curve of the developed model, DMCSH, 2020–2022

The developed model (model) has highest net benefit ratio starting from threshold probability > 0.01 compared to not treating all (horizontal black line) and treating all regardless of their risk (vertical line nearly red lines). The model has the highest net benefit ratio, which has more clinical and public health importance. Therefore, primary attention should be given based on the prognosis model was a higher cost-benefit ratio than not treating all or treating to all regardless of the prediction probability (Fig. 6).

## Discussion

This study provides evidence that age, time of ANC initiation, pregnancy status, recent pregnancy complications, HGB, and parity can predict preterm birth in pregnant women who had ANC visits. We quantified the model’s predictive performance using readily available maternal characteristics during pregnancy rather than invasive laboratory and imaging modalities to guide early interventions to reduce the incidence of preterm birth and improve neonatal health and survival in resource-strained settings such as Ethiopia. It would be used to stratify pregnant women who are at high and low risk for preterm birth and to provide additional management and take appropriate measures accordingly.

The combined maternal characteristics used to predict the risk of preterm birth were age, timing of ANC initiation, pregnancy status, recent pregnancy complications, HGB, and parity. They were significant predictors retained in the final reduced model. This score is built up with predictors, widely available in current practice, simple to investigate, and quite affordable. All of these parameters had already been proven to be predictors of preterm birth in previously published studies.

Age < 20 had significant association with the preterm birth the finding supported by studies conducted in East Africa [46], France [47], India [48], Spain [49], Nairobi [50], and Addis Abeba [18]. The highest risk for preterm birth for teenagers might be related to the fact that most teenage girls may not be mature enough for pregnancy and childbearing. On the other hand, pregnancies in this age group are usually unplanned or unintended, which could result in less interest and fewer chances for mothers to seek prenatal and antenatal care [51]. Besides, young women are more exposed to many risky behaviors like substance use and have less adherence to counselling and education given by their healthcare providers compared to older women. These conditions could have led to a preterm birth [51, 52]. We found that unplanned pregnancy is another significant predictor of preterm birth. The finding supported by studies is that unplanned pregnancy overall is associated with significantly higher rates of preterm birth as compared to a planned pregnancy [1, 53].

Babies born to mothers who seek late initiation of ANC also had a higher risk of being preterm. Other studies have shown that seeking ANC visits later in pregnancy can increase the risk of preterm births [54, 55]. It is also supported by another study that suggests late ANC initiation may lead to poorer outcomes, such as preterm birth, which is very important in detecting early signs of complications and was alarmingly low [56]. Timely and accurate antenatal screening is believed to be an important factor in preventing preterm birth. Antenatal screening also has a positive influence in detecting some pregnancy complications, and this can trigger the offer of perinatal treatment to improve the outcomes and prognosis for preterm infants [57].

Mothers with recent pregnancy complications are a significant predictor of experiencing preterm birth. This finding was consistent with different findings reported that having recent pregnancy complications was a higher risk for preterm birth than not having a recent pregnancy complication (18, 20, 24–26, 34, 37, 42, 51, 58, 62).

Hemoglobin < 11 g/dl (anemia) during recent pregnancy was also another factor for preterm birth. Similarly, findings from the Amhara region [24], California [58], India [59], Nigeria [31], Jimma [22], Wollega [60], Shire [23] and Debretabor [61]. This could be explained biologically by anemia (< 11gm/dl) which causes hypoxia and can induce maternal and fetal stress, which stimulates the production of the corticotrophin-releasing hormone and leads to the initiation of preterm labor. This might also be due to the poor nutritional status of the mother and not having additional meals during pregnancy, which might cause micronutrient deficiency during pregnancy that has been shown to have serious implications for fetal outcomes. As a result, mothers with anemia or low hemoglobin were more likely to have preterm births (23).

Grand parity women and multiparity mothers were at greater risk for preterm delivery compared to null parity. This finding is supported by studies done in India, Nairobi, Kenyatta, and Nigeria [31, 48, 50, 62]. Increasing parity is likely to increase the risk of preterm delivery due to uterine changes such as myometrium stretching from previous pregnancies [32].

The discriminating ability of the developed model for the risk of preterm birth among pregnant women was excellent [63]. The model had discriminative power with an AUROC of 0.931 based on the coefficient (β), and with internal validation (bias-corrected), the performance was 0.928. Based on the risk score, the performance predictive model was 0.926. That means its ability to discriminate against pregnant women who are at higher and lower risk for preterm birth was 92.6% using a simplified risk score.

The model’s accuracy is consistent with a prospective cohort study in European to predict preterm birth results, shows combination of vaginal fluid fetal fibronectin (quantitative fFN) and clinical risk factors had AUROC (0.89) [64]. But predictors necessitate laboratory testing, which is often unavailable in low-resource settings. As a result, such predictors are difficult to come by in ordinary clinical and public health practice, making the model less useful. Other studies, conducted for the prediction of preterm birth in Bahir Dar with performance AUROC of 0.786 [65], study conducted for risk scoring for delivery before 37 weeks of gestation AUC of 0.73 (50) and other retrospective study done in China that established a preterm birth prediction model based on maternal characteristics with AUC of 0.749 [66]. Even if, the performance of those developed model was good, it was lower than our model due to less precise estimation. This might be due to predictors used for model development had less contribution for risk discriminating ability and resulting overfitting. And also inclusion criteria: all of them included twin pregnancies.

The internal validation was done with 2,000 random bootstrap samples with replacements were drawn from the data set with complete data on all predictors. The model’s predictive performance after bootstrapping was excellent [63], with a validation performance of AUROC 0.928. There was a study conducted in France for the prediction of preterm birth to validate the model by sample-split technique, and the performance in validation was lower than the development performance (sample size was 95 vs. 263) [67]. The reason might be that the sample split has limitations due to being unable to quantify the optimism coefficient and a small sample size with many predictors that result in overfitting of model performance. But our model was validated by a bootstrapping validation technique that measures the performance by quantifying the optimism coefficient and also with adequate sample size results.

The performance of our model was also evaluated in terms of calibration by calibration plot and statistical test. That showed insignificant statistical test by Hosmer-Lemshow (p-value = 0.066) test and well calibrated (calibration plot falling along a 45° line) [43], meaning the degree of agreement between observed proportions of preterm birth and predicted probabilities of preterm birth was good and no misrepresentation of the data, which shows its reliability.

The simplified risk score developed from the regression model is easier to use in routine clinical and public health practice and had comparable discrimination power and well calibrated. In our prediction score, using 4 as the cutoff point has an acceptable level of specificity (82.1%), sensitivity (91.0%), and accuracy (83.1%) to predict preterm birth. Depending on the aim of the program and the availability of resources, it is also possible to change the cutoff point to increase either of the accuracy measures. A prospective cohort study was conducted in India to develop an antenatal risk scoring system/scale for the prediction of preterm birth. The study stated that the developed scoring system resulted in good sensitivity at a cutoff point of ≥ 8 of 75.5% [68]. Another study done in Bahir Dar threshold score to predict preterm birth using risk scores is ≥ 3, with a sensitivity of 75.14% and a specificity of 67.46% [65]. Those findings have low sensitivity as compared to our study, which might cause maximizing the number of false negatives, which results in an unreliable way to rule out a higher likelihood of having a preterm birth. And also, false negatives will be higher, which have a higher impact on preterm birth prevention due to the seriousness of the disease.

The developed prognosis risk score results in an AUC of 0.926, which is excellent accuracy according to the diagnostic accuracy classification. The prediction accuracy to differentiate pregnant mothers who had a high and low risk of preterm birth is 92.6%, which is determined by the predictors. It is useful to know what proportion of women who go on to experience preterm birth are identified by risk scoring as being at high risk (sensitivity of the test) and what proportion of those who do not go on to experience preterm birth are identified by the test as being at low risk (specificity).

We used decision curve analysis (DCA) to assess the model’s clinical and public health impact. Despite the fact that model performance was evaluated in terms of discrimination and calibration, it was satisfactory. In a wide range of threshold probabilities, the decision curve analysis revealed that the developed risk score for early categorization of preterm birth risk has greater clinical benefit than treating none or treating all pregnant mothers. The decision curve analysis concept is standard net benefit along threshold probability. The standard net benefit is calculated by subtracting the benefit from the cost. In the case of this study, the benefit is treating true positives (after correctly predicting pregnant women will have preterm birth), while the cost is treating false positives (incorrectly predicting pregnant women will experience preterm birth).

The net benefit of the model-based treatment decision is greater than the net benefit of treating none (assume no women have preterm birth) or treating all (assume all women have preterm birth). The developed model is compared to two extreme scenarios: “treating all” women results in unnecessary interventions for those at low risk or involves the expenditure of time, effort, and money, and " treating none " results in a lack of intervention for those high-risk mothers, leading to increased prematurity and its associated complications regardless of risk. Our model assumes that pregnant women who are at higher risk of preterm birth are treated by providing high-level antenatal care aimed at preventing preterm birth in those identified as being at increased risk, corticosteroid administration, antibiotic treatment in the event of infection, or by transferring them in utero to a hospital with neonatal intensive care available.

### Strength and limitation of the study

It was conducted with an adequate sample size (adequate number of participant outcomes) for predicting preterm birth, which helped construct the model using a sufficient number of candidate predictors and protect overfitting. The model was developed based on easily accessible and measurable maternal characteristics that can be applied in any clinical setup. Moreover, it was validated internally, and the optimism coefficient was minimal, indicating that it is less likely that the model is sample-dependent.

The study was not without limitations, in retrospectively collected data, some variables for the prediction of preterm birth might have been missed. However, models developed using retrospectively collected data are still important in resource-limited settings like Ethiopia. However, internal validation was done, It would have been better if it had gone through external validation to ensure its prediction capability when applied to other contexts.

### Clinical practice implications

The developed risk score model is easy for clinical application, including predictors that can be easily accessed with no advanced clinical assessment of pregnant women. The prediction score will help with the risk stratification of pregnant women and identify those at higher risk of preterm birth. Subsequently, high-risk groups can be given special attention and care, or we can early identify women who are at higher risk of premature delivery so that we can offer prophylactic interventions and guide antenatal management decisions. The women’s categorization into low and high-risk groups provides optimized sensitivity and specificity. Clinicians are believed to be better informed by calculating the risk estimate using an appropriate cut-off for each pregnant woman and making decisions based on conditions.

Our prediction model includes variables that are easily obtained and have a high enough accuracy to be used by both mid-level and lower-level health professionals in primary care settings. Five of the maternal characteristics included in our model can be easily identified through history-taking and one through hemoglobin testing. As a result, this feasible prognosis risk score would provide a chance to reduce neonatal complications associated with prematurity, thereby improving overall maternal and child healthcare. The model is critical to preventing the devastating personal, economic, and health consequences of preterm birth.

## Conclusions

This study shows the possibility of predicting preterm birth using a simple prediction model constructed from maternal characteristics. Thus, the optimal combination of maternal characteristics such as age (< 20), late initiation of ANC, unplanned pregnancy, recent pregnancy complications, multiparty, and hemoglobin < 11 mg/dl showed the possibility of predicting preterm birth. In addition, risk score calculations based on a combination of predictors were effective and had comparable accuracy with the model-based approach of the original β coefficients. It can be used in the clinical settings by healthcare providers for early detection, timely decision making, and improving care quality. They can then save lives.

### Electronic supplementary material

Below is the link to the electronic supplementary material.

**Supplementary Material 1**: **Supplementary Table 1**. Bivariable logistic regression analysis of predictors of preterm birth among pregnant women who had ANC visit at DMCSH, 2020–2022

## Data Availability

The data sets used and analyzed during the current study was available from the corresponding author on reasonable request.

## Abbreviations

ACOG: American College of Obstetricians and Gynecologists
ANC: Antenatal Care
AUROC: Area Under Curve of the Receiver Operating Characteristic
CI: Confidence Interval
DCA: Decision Curve Analysis
DMCSH: Debre Markos Comprehensive and Specialized Hospital
EDHS: Ethiopia Demographic Health Survey
HGB: Hemoglobin
HIV: Human Immune Virus
HSTP-II: Health Sector Transformation Plan II
ICU: Intensive Care Unit
GA: Gestational Age
LMIC: Low and Middle Income Country
LNMP: Last Normal Menstrual Period
MUAC: Middle Upper Arm Circumference
NPV: Negative Predictive Value
PIH: Pregnancy Induced Hypertension
PPV: Positive Predictive Value
PROM: Premature rapture of membrane
PTB: Preterm Birth
ROC: Receiver Operating Characteristic
TRIPOD: Transparent Reporting of a multivariable prediction model for Individual Prognosis Or Diagnosis
WHO: World Health Organization
