# Supplementary material for: Development and validation of a prognosis risk score model for preterm birth among pregnant women who had antenatal care visit, Northwest, Ethiopia, retrospective follow-up study
Source: BMC Pregnancy Childbirth. 2023 Oct 17;23:732. doi: 10.1186/s12884-023-06018-1 (PMC10583360; doi:10.1186/s12884-023-06018-1)
Supplement: Supplementary file 1 — Supplementary Material 1: Supplementary Table 1. Bivariable logistic regression analysis of predictors of preterm birth among pregnant women who had ANC visit at DMCSH, 2020–2022 [file 12884_2023_6018_MOESM1_ESM.docx]

**Supplementary Table 1****:** Bivariable logistic regression analysis of predictors of preterm birth among pregnant women who had ANC visit at DMCSH, 2020- 2022.

| **Predictor Variable** | **Category** | **Preterm birth** | | **β (95%CI)** | **p-value** |
| --- | --- | --- | --- | --- | --- |
|  |  | Yes | No |  |  |
| Age | <20 | 18 | 21 | 2.246(1.54 ,2.952) | 0.001 |
|  | 20-24 | 13 | 222 | 1 |  |
|  | 25-29 | 41 | 452 | -0.438(-1.082,0.207) | 0.183 |
|  | 30-34 | 25 | 206 | 0.291(-.233,0 .815) | 0.276 |
|  | ≥35 | 26 | 108 | 0.976(.442,1.51) | <0.001 |
| Residency | Urban | 91 | 895 | 1 |  |
|  | Rural | 32 | 114 | 1.016(.568 1.463) | 0.001 |
| Marital | Married | 118 | 984 | 1 |  |
|  | Single | 3 | 18 | 0.329(-.908,1.566) | 0.602 |
|  | Divorced/widow/  Separate | 2 | 7 | 0.868(-.715, 2.451) | 0.282 |
| MUAC | ≥24cm | 77 | 793 | 1 |  |
|  | <24 cm | 46 | 216 | 0.785(0.39, 1.18) | 0.001 |
| Weight of Mother | ≥ 50kg | 96 | 840 | 1 |  |
|  | < 50 kg | 27 | 169 | 0.335(-0.123, .793) | 0.152 |
| Gravidity | Primigravida | 33 | 377 | 1 |  |
|  | Multigravida | 90 | 612 | 0.487(0.068,0.905) | 0.023 |
| Parity | Nullpara | 39 | 623 | 1 |  |
|  | Primipara | 26 | 287 | 0.018(-0.536, 0.501) | 0.947 |
|  | Multipara | 58 | 299 | 0.744(0.312, 1.176) | 0.001 |
| Pregnancy status | Planned | 37 | 715 | 1 |  |
|  | Unplanned | 86 | 294 | 1.73(1.324, 2.141) | 0.001 |
| Timing of ANC initiation | Early initiation | 14 | 271 | 1 |  |
|  | Late initiation | 109 | 738 | 1.05(0.477, 1.624) | 0.001 |
| Time of initiating iron folate | 1st trimester | 34 | 232 | 1 |  |
|  | 2nd trimester | 86 | 747 | -.241(-0.665,0.182) | 0.264 |
|  | 3rd trimester | 3 | 30 | -.382(-1.622, 0.858) | 0.546 |
| Rh status | Positive | 117 | 934 | 1 |  |
|  | Negative | 6 | 75 | -.448(-1.302, 0.405) | 0.303 |
| Hemoglobin | ≥11g/dl | 83 | 997 | 1 |  |
|  | <11 g/dl | 40 | 12 | 3.69(3.007, 4.373) | 0.001 |
| Comorbid | No | 118 | 990 | 1 |  |
|  | Yes | 5 | 19 | 0.792(-0.211, 1.795) | 0.122 |
| HIV result | Negative | 119 | 958 | 1 |  |
|  | Positive | 4 | 51 | -.46(-1.495, 0.576) | 0.384 |
| Recent pregnancy complication | No | 41 | 941 | 1 |  |
|  | Yes | 82 | 68 | 3.321(2.872, 3.769) | 0.001 |

**MUAC Middle Upper Arc Circumference, *ANC Antenatal care, *CI confidence interval*
